# BRAF associated autophagy exploitation: BRAF and autophagy inhibitors synergise to efficiently overcome resistance of BRAF mutant colorectal cancer cells

**DOI:** 10.18632/oncotarget.6942

**Published:** 2016-01-18

**Authors:** Maria Goulielmaki, Evangelos Koustas, Eirini Moysidou, Margarita Vlassi, Takehiko Sasazuki, Senji Shirasawa, George Zografos, Eftychia Oikonomou, Alexander Pintzas

**Affiliations:** ^1^ Laboratory of Signal Mediated Gene Expression, Institute of Biology, Medicinal Chemistry and Biotechnology, National Hellenic Research Foundation, Athens, Greece; ^2^ Institute for Advanced Study, Kyushu University, Fukuoka, Japan; ^3^ Department of Cell Biology, Faculty of Medicine, Fukuoka University, Fukuoka, Japan; ^4^ 3rd Department of Surgery, General Hospital of Athens G. Gennimatas, Athens, Greece

**Keywords:** colorectal cancer, BRAF inhibitors, autophagy inhibitors, synergistic treatments

## Abstract

Autophagy is the basic catabolic mechanism that involves cell degradation of unnecessary or dysfunctional cellular components. Autophagy has a controversial role in cancer – both in protecting against tumor progression by isolation of damaged organelles, or by potentially contributing to cancer growth. The impact of autophagy in RAS induced transformation still remains to be further analyzed based on the differential effect of RAS isoforms and tumor cell context. In the present study, the effect of KRAS/BRAF/PIK3CA oncogenic pathways on the autophagic cell properties and on main components of the autophagic machinery like p62 (SQSTM1), Beclin-1 (BECN1) and MAP1LC3 (LC3) in colon cancer cells was investigated. This study provides evidence that *BRAF* oncogene induces the expression of key autophagic markers, like LC3 and BECN1 in colorectal tumor cells. Herein, PI3K/AKT/MTOR inhibitors induce autophagic tumor properties, whereas RAF/MEK/ERK signalling inhibitors reduce expression of autophagic markers. Based on the ineffectiveness of BRAFV600E inhibitors in BRAFV600E bearing colorectal tumors, the BRAF related autophagic properties in colorectal cancer cells are further exploited, by novel combinatorial anti-cancer protocols. Strong evidence is provided here that pre-treatment of autophagy inhibitor 3-MA followed by its combination with BRAFV600E targeting drug PLX4720 can synergistically sensitize resistant colorectal tumors. Notably, colorectal cancer cells are very sensitive to mono-treatments of another autophagy inhibitor, Bafilomycin A1. The findings of this study are expected to provide novel efficient protocols for treatment of otherwise resistant colorectal tumors bearing BRAFV600E, by exploiting the autophagic properties induced by *BRAF* oncogene.

## INTRODUCTION

The PI3K/AKT/MTOR and RAF/MEK/ERK signalling pathways are involved both in normal cell properties like regulation of cell proliferation and survival, as well as in the development of cancer [1]. Mutations within *RAS*, *BRAF* and *PI3K* oncogenes have been frequently detected in colorectal cancer. The most frequent BRAFV600E mutation is a single substitution at nucleotide 1796. The phosphoinositide 3-kinase (PI3K) is another well-studied RAS effector. PI3K family members play an important role as mediators of RAS-regulated cell survival and proliferation. When PI3K is active, it can trigger cell growth, cell cycle entry, and/or cell survival through phosphorylation of AKT [1, 2]. Most KRAS and BRAF mutations enhance their ability to directly phosphorylate MEK. The role of RAS and PI3K signalling pathways has been analysed in regulating the autophagic process in different systems, although key mechanisms are still under investigation.

Autophagy is a housekeeping survival mechanism with a protective function against stress conditions where the cells start to digest their own cellular components [3]. In tumors, this self-cannibalization process is stimulated by metabolic stress (e.g., nutrient/growth factor deprivation, hypoxia, and acidosis), cellular damage, or inhibition of pro-survival signals caused by anticancer therapies [4]. Through autophagy cancer cells utilize a highly plastic and dynamic mechanism to either repress initial steps of carcinogenesis, or support the survival and growth of established tumors [5, 6].

BECN1, rarely mutated in tumors [20], and LC3 proteins are two key components of the autophagic process. Precisely, LC3-BI is converted to LC3-BII through lipidation by an ubiquitin-like system involving ATG7 and ATG3 that allows LC3 to become associated with autophagic vesicles. p62 (SQSTM1) is thought to be another critical protein that targets other proteins for proteasome degradation or autophagic digestion, at the crossroads of autophagy, apoptosis and cancer. In particular, LC3-II binds p62 to regulate protein packaging and delivery to the autophagosome [3]. Both the presence of LC3 in autophagosomes and the conversion of LC3 to the lower migrating form, LC3-II, have been used as indicators of autophagy [3, 7]. Mammalian BECN1, also known as autophagy-related gene (*ATG6*), has been shown to interact with BCL-2 and BCL2L1 [8]. Furthermore, BECN1 and its binding partner PIK3C3, also known as Vps34, are required for the initiation of the autophagosome formation. On the other hand, the MTOR signaling pathway downstream of AKT, is a critical regulator of autophagy. Activation of MTOR can inhibit the autophagic process [9].

Previous studies propose that mutant *RAS* oncogene can prevent the autophagophore formation through downregulation of BECN1 and thus, can promote the anchorage-independent growth of malignant cells through a mechanism that involves down-regulation of BECN1 [10]. In other studies, mutant HRAS has been shown to induce autophagic traits [11]and cell death through autophagy or RAS was shown to promote autophagic cell death [12]. Other studies argue that hypoxic regions of established tumors by activated RAS present high autophagic activity, through which cells can survive under stressful conditions [13]. Most important, the putative association of activated BRAF to autophagy has not yet been analyzed in detail.

The hypothesis of the controversial role of autophagy in tumorigenesis and survival is supported in a number of studies [19]: while some studies have suggested a tumor suppressive role for autophagy [14–16] and the inactivation of autophagy-related genes in certain human cancers [17], others have reported the opposite. According to this, tumor cells were found to exhibit high basal levels of autophagy required for cancer cell proliferation [13, 18].

Recent developments in the investigation of autophagy as a mechanism of resistance to many anticancer drugs and the development of strategies to inhibit autophagy represent a new approach to enhance the efficacy of cancer therapy [21]. Current inhibitors of autophagy, like 3-MA, Bafilomycin, (hydroxyl) chloroquine as well as new agents-modulators of autophagy, are currently tested as anti-cancer agents, either alone, or in rational combinatorial treatments with targeted anti-cancer drugs [22]. These recent findings confirm the urgent need for further investigation into the relation between autophagy, tumorigenesis and tumor survival, since these may also be dependent on the cell type, tumor stage and environmental conditions [23].

The specific BRAFV600E inhibitors vemurafenib [27] and dabrafenib accomplish relatively short periods of antitumor activity before the appearance of resistance and treatment failure. A number of different mechanisms that may contribute to tumor resistance to drug therapy have been proposed, including a feedback re-activation of EGFR in colorectal cancer [24-26, 28-30]. Several studies and clinical trials in various cancer types have long been focusing on combining specific BRAFV600E inhibitors with other kinase or receptor growth factor inhibitors, like MEK, PI3K and EGFR [31-33, 44]. Towards this end, combined inhibition of autophagy and oncogenic BRAF may be particularly efficient, as BRAF-driven tumors induce and require mitochondrial metabolism, and therefore may need autophagy to supply substrates, such as glutamine, to replenish TCA cycle intermediates and to eliminate damaged mitochondria [34].

In the present study, the role of autophagy in RAS and BRAF induced transformation was examined in colon cancer cell lines. Both stimulation of LC3 expression and formation of autophagic vacuoles in mutant BRAFV600E cell lines are presented. Furthermore, the MEK/ERK pathway can increase the protein levels of LC3, unlike the AKT/MTOR pathway, which has been shown to abolish the autophagic process. Notably, Bafilomycin A1, a potent autophagy inhibitor can cause remarkable apoptotic cancer cell death, as mono-treatment. It is shown here that by using specific autophagy inhibitors not only cancer cell proliferation rate can be reduced, but the otherwise resistant mutant BRAF colon cell lines to targeted BRAF agents, like PLX4720 (Vemurafenib), can be sensitised to apoptosis in a synergistic manner. This study proposes a promising rational combinatorial treatment using BRAF and autophagy inhibitors that will potentially provide efficient therapeutic protocols for these otherwise untreatable tumors, yet to be further tested *in vivo*.

## RESULTS

### Steady levels of autophagic markers in basic colon adenocarcinoma cell lines

**Figure 1:**
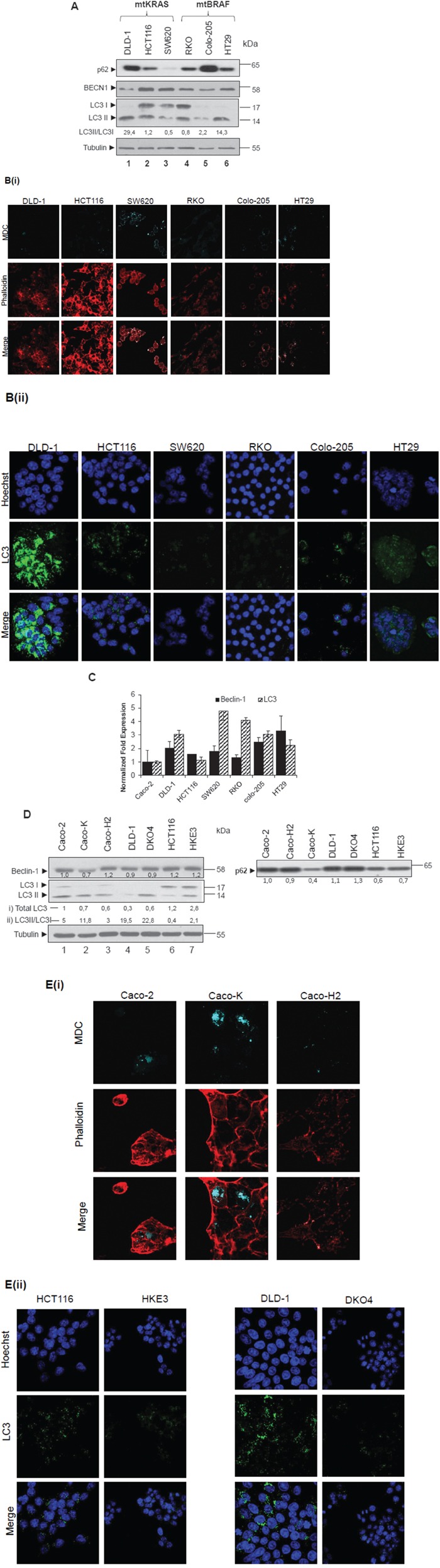
Steady state levels of autophagic markers LC3 and BECN-1 in colon cancer cell lines **A-E.** Steady protein (A, lanes 1-6) and mRNA levels (C) of autophagic markers in basic colon adenocarcinoma cell lines (DLD-1, HCT116 SW620, RKO, Colo-205 and HT29); also in cell lines derived after over-expression or down-regulation of mutant RAS (Caco-K, Caco-H2, DKO4 and HKE3) (D, lanes 1-7). Caco-2, as intermediate adenoma cell line, is used as control. Using Western blot assay, the protein levels of the two autophagic markers BECN1 and LC3, as well as those of p62 were analyzed in the above cell lines (A, D). For LC3 and BECN1, mRNA levels were also analyzed, using the Real-Time PCR assay. The autophagic vacuoles were detected with 0,1 mM of MDC (light blue) under confocal microscope, while phalloidin staining (red) was used for detection of the cell number in the corresponding cell lines (B(i), E(i)). Staining was also performed using Hoechst (B(ii),E(ii), upper row, blue), LC3 antibody (B(ii),E(ii), middle row, green) and merged (B(ii),E(ii) lower row). The quantification of LC3 reflects the ratio LC3II/LC3I in 1A, while in 1D both total LC3 levels as compared to Caco-2 cells (i) and LC3II/LC3I ratio (ii) have been recorded. Data are representative for three independent experiments. Protein levels were normalized against tubulin. mRNA levels were normalized against GAPDH. Standard Deviation (SD) was calculated automatically by software and the value is shown as a bar in the histograms.

Different colon adenocarcinoma cell lines were examined with respect to autophagic properties. In all cell lines, presence of autophagy was tested through the relative levels of LC3II using western blot (Figure 1A, lanes 1-6) and then confirmed with MDC staining- a molecule that stains autophagic vacuoles. Phalloidin was used for cytoskeleton staining, in order to assess the number of cells present in each sample (Figure 1B(i)). A strong association between LC3II and p62 (SQSTM1) expression and MDC positive staining of autophagic vacuoles in colon cancer cell lines was revealed. In detail, protein levels of the autophagic marker LC3 (I and II) were found elevated in DLD-1 (lane 1), HCT116 (lane 2) and RKO (lane 4) cells, while lower was the expression in SW620 (lane 3) and Colo-205 (lane 5) adenocarcinoma cell lines. Furthermore, LC3 staining revealed also high protein distribution in DLD-1 (Figure 1B(ii)). The ratio between LC3II and LC3I protein levels was notably higher in DLD-1 and HT29 as compared to the other cell lines. The levels of BECN1 were found elevated only in HCT116 and SW620 cell lines, whereas the ratio between LC3II/LC3I was low. The flux of autophagy was also examined through Western blot analysis of p62 protein levels, which were found elevated in DLD-1 and Colo-205 and reduced in SW620 and HT29 cells, as compared to the other cell lines (Figure 1A). Alongside, mRNA level analysis, showed higher than 3-fold mRNA LC3 expression in DLD-1, Colo-205, SW620 and RKO, as compared to Caco-2 intermediate colon adenoma cells. The mRNA levels of *BECN1* were found increased by at least 2-fold in DLD-1, SW620, Colo-205 and HT29 as compared to Caco-2 (Figure 1C). Notably, RKO, HCT116 and SW620 cell lines exhibit properties of Epithelial-Mesenchymal-Transition (EMT) phenotype. These results propose a putative relation between autophagy and the EMT cell programs.

Therefore, the protein levels of autophagic markers BECN1 and LC3 are overexpressed in selected colon adenocarcinoma cell lines. HT29 and SW620 cell lines show induced autophagic properties as revealed by high MDC staining and low p62 protein levels.

### *KRAS* and *HRAS* oncogenes differentially regulate the autophagic markers p62, LC3 and BECN1

Since autophagic markers are differentially expressed in colorectal cell lines bearing *KRAS* or *BRAF* mutations, as shown in Figure 1A, the impact of either *KRAS* or *BRAF*, as well as that of *HRAS* oncogenes on autophagy was further examined. In Caco-K cell line (Figure 1D, lane 2), generated after stable ectopic over- expression of KRAS oncogene in Caco-2 cells [36], the protein levels of autophagic markers BECN1 and LC3 are lower in comparison to the parental Caco-2 cells. On the contrary though, in Caco-K cells the ratio between LC3 II and LC3I is elevated, while p62 protein levels are reduced, indicating an over-activation of the autophagic machinery, as compared to the parental Caco-2 cells. As a further confirmation for the induction of autophagy, the number of MDC stained vacuoles increased (Figure 1E(i), 2nd column). On the other hand, in Caco-H2 cell line generated after stable over-expression of *HRAS* oncogene in Caco-2 cells, BECN1 expression was 20% higher and the total amount of LC3 was lower as compared to parental Caco-2 cells. The ratio between LC3II and LC3I was also lower, while p62 protein levels were not affected (Figure 1D) by *mtHRAS*. Furthermore, in DKO4 (lane 5) and HKE3 (lane 7) cell lines (generated after specific knock-out of mt*KRAS* in DLD1 (lane 4) and HCT116 (lane 6) cells respectively), LC3II levels were higher than parental DLD-1 and HCT116 cells respectively, but LC3B staining was reduced in these cell lines (Figure 1E(ii)). p62 and BECN1 protein levels (Figure 1D), as well as the number of MDC stained autophagic vacuoles did not change significantly (Data not shown). Thus, deletion of activated *KRAS* oncogene did not affect the autophagic properties of the cells, as detected by western blot and immunofluorescence. The fact that the ratio between LC3II/LC3I was low in HCT116 and Caco-H2, two cell lines with EMT phenotype, contributes to the hypothesis of a potential correlation of EMT and autophagy (Figure 1D).

These data suggest that KRAS over-expression induces the autophagic properties in colorectal cancer cells. It is also evident that KRAS and HRAS oncogenes differentially regulate autophagic cell properties in the examined colorectal cancer cells.

### BRAFV600E induces the expression of autophagic markers BECN1 and LC3. Association of BRAFV600E with autophagy

**Figure 2:**
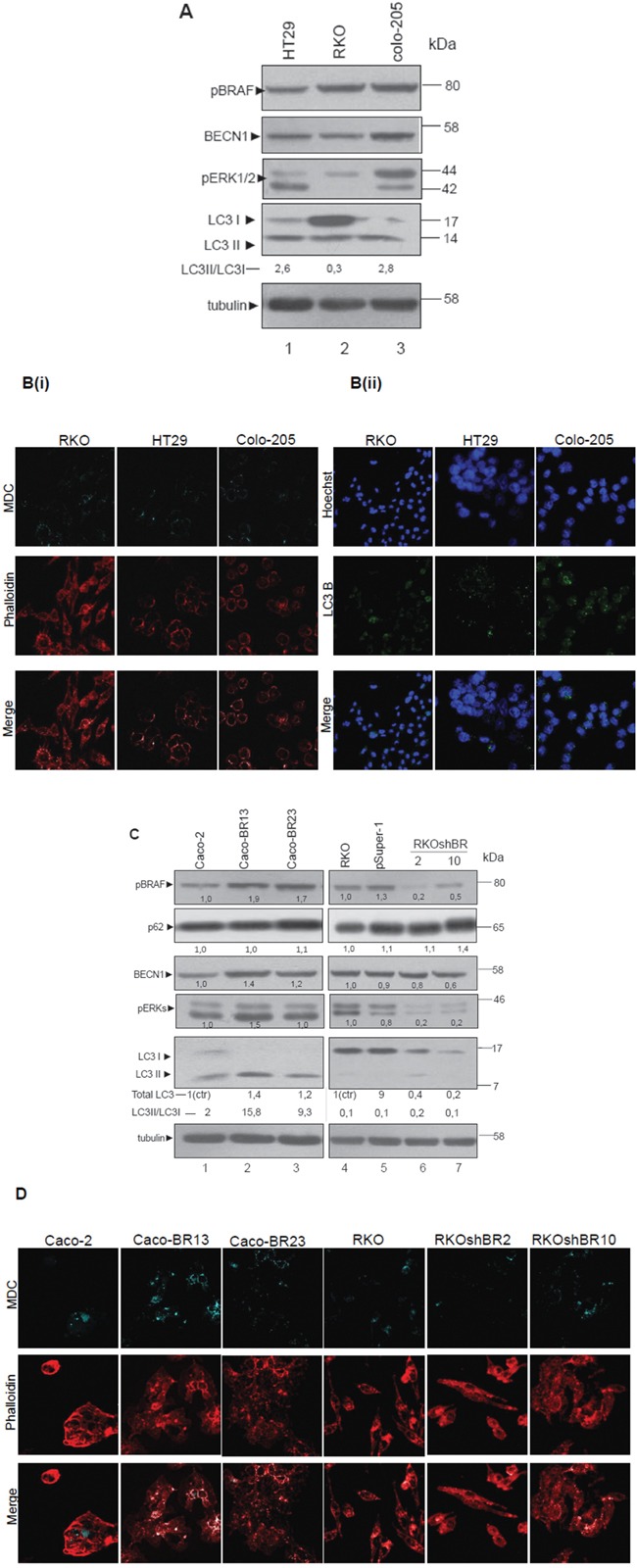
BRAF oncogene induces the expression of LC3- Association of autophagy with mutant BRAFV600E **A-D.** Steady protein levels of autophagic markers LC3 and BECN1, as well as p62 in colon adenocarcinoma cell lines with mtBRAFV600E (RKO, HT29 and Colo-205) (A, lanes 1-3) and in cell lines with over-expression (Caco-BR13, Caco-BR23) (C, lanes 1-3), or down-regulation of mutant BRAFV600E (RKOshBR 2, and RKOshBR 10) (C, lanes 4-7). Control cell lines were Caco-2 cell line for Caco-BR cell lines-clones and RKO cell line for RKOpsuper-1 and RKOshBR cell lines-clones. Quantification of LC3 for Caco-BR clones reflects the whole protein levels as compared with Caco-2 or for RKOshBR and RKOpsuper-1 clones compared with RKO (i) and the ratio of LC3II/LC3I in both cases (ii). Protein levels were normalized against tubulin. The autophagic vacuoles were detected with 0,1 mM of MDC (light blue) with confocal microscopy, while phalloidin staining (red) was used for detection of the cell number in each sample (B(i) and D). Staining was also performed using Hoechst (2B(ii), upper row, blue), LC3 antibody (2B(ii), middle row, green) and merged (2B(ii), lower row). Data are representative for three independent experiments.

In RKO and Colo-205 cell lines bearing BRAFV600E, protein levels of the autophagic markers BECN1 and LC3 were high. Activity of MEK/ERK signalling pathway was also shown to be associated to the expression of these autophagic markers (Figure 2A, lanes 1-3). As an additional confirmation, MDC staining revealed the presence of autophagic vacuoles in a high percentage of phalloidin stained cells (Figure 2B(i)). Staining of these cell lines with LC3 antibody under confocal microscope, showed a high number of dispersed LC3 punkta around the nucleus, which were more intense in Colo-205 (Figure 2B(ii)). Thus, presence of autophagy was obvious in all three BRAFV600E cell lines.

In order to further analyze the putative induction of autophagy by BRAFV600E, cell lines stably over- expressing BRAFV600E in Caco-2 cells (Caco-BR13 and Caco-BR23) were examined (Figure 2C, lanes 1-3). BRAFV600E induced MEK/ERK signalling pathway activity in Caco-BR compared to the parental Caco-2 cells and was associated with elevated protein expression levels of autophagic markers BECN1 and LC3, in particular of LC3II, as shown by Western blot (W.B.) analysis (Figure 2C, lanes 1-3). Additional evidence for the role of BRAFV600E in autophagy was provided from parallel experiments: In RKOshBR2 and RKOshBR10 cells developed after silencing of BRAFV600E in RKO cell line, MEK/ERK signalling pathway was suppressed and the protein levels of autophagic markers BECN1 and LC3 were decreased by W.B. analysis, as compared to parental RKO (Figure 2C, lanes 4-7). Furthermore, p62 protein levels were found increased in RKOshBR10 cell line. In all cell lines, changes in autophagy were shown through LC3II/LC3I ratio by W.B. analysis (Figure 2A, C) and were further confirmed with MDC staining shown by confocal microscopy (Figure 2B(i), 2D). In general, in BRAFV600E cell lines with high LC3II/LC3I ratio, more MDC stained vacuoles were detected (Figure 2D). These results reveal a possible interaction between BRAFV600E induced tumor properties, MEK signalling and autophagy. These findings were examined in BRAFV600E colorectal cancer specimens. Notably, three out of four tumor (75%) clinical samples bearing BRAFV600E have shown high mRNA expression of *BECN1* and *LC3* with respect to the matched normal tissue (Table 1). This preliminary evidence should be further validated with a larger number of samples.

**Table 1:** BECN1 and LC3 are overexpressed in BRAFV600E colorectal adenocarcinomas Real-time analysis of human colorectal cancer samples for *BECN1* and *LC3* mRNA

BRAFV600E Tissue Sample	Gene	mRNA Overexpression Fold
**1**	BECN1	0,82
	LC3	1,45
**2**	BECN1	1,44
	LC3	1,30
**3**	BECN1	1,13
	LC3	0,83
**4**	BECN1	2,43
	LC3	2,12

For genes mRNA expression in tumor tissues has been normalized using as reference sample a pool of normal tissues and normal expression was set to 1.

Data provided here indicate that BRAFV600E induces high expression of autophagic markers and further suggest that BRAFV600E regulated MEK/ERK signalling may provide colon cancer cells with autophagic properties.

### The MEK/ERK signalling pathway positively regulates autophagic markers. Induction of autophagy requires simultaneous inhibition of AKT and MTOR

**Figure 3:**
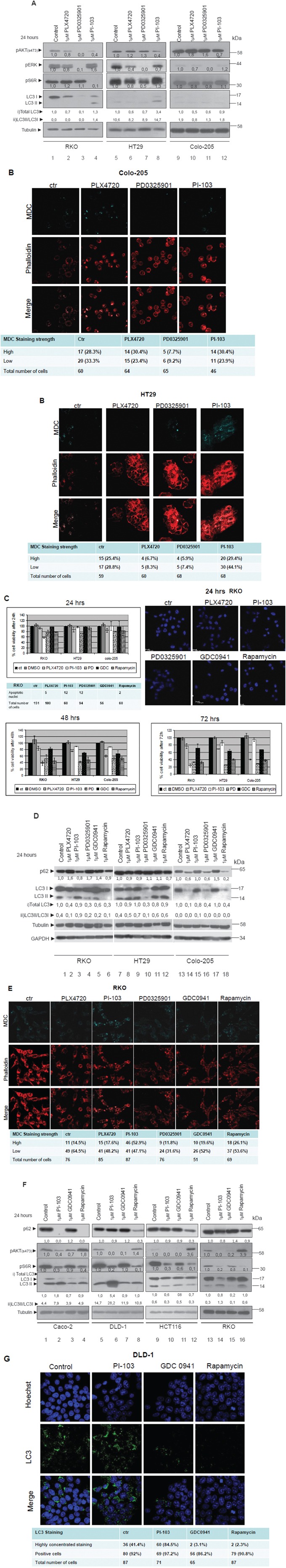
Differential regulation of autophagy by the two signalling pathways MEK/ERK and AKT/MTOR **A.** Western blot analysis of protein levels of pAKT (ser473), pS6R, pERKs, LC3 and tubulin after 24 h treatment with 1μM BRAFV600E inhibitor PLX4720 (lanes 2, 6 and 10), 1μM MEK inhibitor PD0325901 (lanes 3, 7 and 11) and 1μM of PI3K/MTOR inhibitor PI-103 (lanes 4, 8, 12), in the mutant BRAFV600E cell lines RKO, HT29 and Colo-205 respectively. The quantification of LC3 reflects the whole protein levels as compared to the untreated sample in each cell line (i) and the ratio of LC3II/LC3I in each sample separately (ii). **B.** The existence of autophagic vacuoles was analysed by 0,1 mM MDC staining under confocal microscope, after treatment of HT29 and Colo-205 cell lines with the kinase inhibitors, described in 3A. **C.** Cell viability of the mutant BRAFV600E colon cancer cell lines RKO, HT29 and Colo-205 after 24 (upper left panel), 48 (lower left panel) and 72 h (lower right panel) treatments with 1μM of each of the following BRAF/MEK/ERK and AKT/MTOR pathway inhibitors; PLX4720, PI-103, PD0325901 (PD), GDC0941 (GDC) (PI3K inhibitor), Rapamycin (MTOR inhibitor). Cells were also stained with Hoechst after 24h treatment in order to assess the number of apoptotic nuclei (upper right panel). **D.** Western blot analysis of LC3 and p62 protein levels in the mutant BRAFV600E colon cancer cell lines RKO, HT29 and Colo-205 respectively after 24 h treatment with 1μM of each of the following BRAF/MEK/ERK and AKT/MTOR pathway inhibitors: PLX4720 (2, 8, 14), PI-103 (3, 9, 15), PD0325901 (4, 10, 16), GDC0941 (5, 11, 17), Rapamycin (6, 12, 18). The quantification of LC3 reflects total protein levels as compared to the untreated sample in each cell line (i) and the ratio of LC3II/LC3I in each sample separately (ii). **E.** Confocal microscope images of two-dimensional culture in RKO cell line after treatment with 1μM of each of the following BRAF/MEK/ERK and PI3K/MTOR pathway inhibitors; PLX4720 (2^nd^ column), PI-103 (3^rd^ column), PD0325901(4^th^ column), GDC0941(5^th^ column), Rapamycin (6th column) as compared to control untreated cells (1st column). Cells were stained with MDC (upper row, light blue), phalloidin (middle row, red), and merged staining (lower row), in order to detect autophagic vacuoles (MDC) and cell number distribution. The number of cells stained with MDC (high-low) was recorded. The total number of cells by phalloidin staining and the number of MDC stained cells from five different confocal images for each sample was recorded. **F.** Western blot analysis of protein levels of pAKT(ser473), pS6R, LC3, p62 and tubulin after 24 h treatment with 1μM of PI-103 (lanes 2, 6, 10, 14), 1μM GDC0941 (lanes 3, 7, 11, 15) and 1μM Rapamycin (lanes 4, 8, 12, 16) in Caco-2, DLD-1, HCT116 and RKO cell lines. Protein levels were normalized against tubulin. The quantification of LC3 reflects the total protein levels as compared to the untreated sample in each cell line (i) and the ratio of LC3II/LC3I in each sample separately (ii). **G.** Confocal microscope images of two-dimensional culture in DLD-1 cell line after treatment with 1μM of each of the following PI3K/MTOR pathway inhibitors; PI-103 (2^nd^ column), GDC0941(3^rd^ column) and Rapamycin (4th column) as compared to control untreated cells (1st column). Cells were stained with Hoechst (upper row, blue), LC3 antibody (middle row, green) and merged (lower row). The total number of cells by Hoechst staining and the number of LC-3 stained cells from five different confocal images for each sample was recorded.

We initially examined the effect of BRAF/MEK and PI3K/MTOR pathway inhibitors on cell signaling and autophagy of BRAFV600E bearing colorectal cancer cell lines (Figure 3A, 3B, 3D), in order to have a first indication on the potential regulation of autophagy by these two pathways. Then, we aimed to detect the role of autophagy on apoptosis by inhibiting either MEK or PI3K: Analysis of cell viability, autophagy and apoptosis after cell treatments with a number of BRAF/MEK and PI3K/MTOR inhibitors on BRAFV600E cell lines was performed (Figure 3C, 3E). Finally, the comparative induction of autophagy by different PI3K/MTOR inhibitors in non-BRAFV600E cell lines was further examined (Figure 3F–3G).

### BRAF/MEK/ERK inhibition

The hypothesis of MEK/ERK regulated autophagy was further tested. MEK/ERK pathway was inhibited in three cell lines with mutant BRAFV600E (RKO, HT29 and Colo-205) after treatment with 1μM of PLX4720 (specific BRAFV600E inhibitor) (Figure 3A, lanes 2, 6, 10) and 1μM of PD0325901 (MEK inhibitor) (Figure 3A, lanes 3, 7, 11) for 24 h. The total number of cells was measured either by phalloidin or by Hoechst staining, as indicated. Treatment with MEK inhibitor PD0325901 markedly reduced MEK/ERK activity in BRAFV600E bearing colon cancer cell lines (Figure 3A, lanes 3, 7, 11), which was accompanied by a remarkable reduction of autophagic markers expression, particularly in LC3II (Figure 3A, 3D). Treatment of BRAFV600E bearing colon cells with PLX4720 had a more profound effect on MEK/ERK activity and autophagic properties than MEK inhibitor treatment (Figure 3A, lanes 2, 6, 10). On the opposite, p62 protein levels were elevated after treatment of RKO with PLX4720 or PD0325901, indicating suppression of autophagy. Remarkably, p62 levels in Colo-205 were reduced after treatment with these BRAF/MEK inhibitors, following the reduction in total LC3 levels (Figure 3D). The inhibition of autophagy through MEK/ERK pathway was also confirmed by reduction in MDC-vacuole-staining after treatment of BRAFV600E colon cancer cell lines with PLX4720 and PD0325901 under confocal microscopy (Figure 3B, HT29 and Figure 3E). Finally, MEK inhibitor treatment resulted in an average 36% reduction of autophagic vacuole formation in RKO (Figure 3E), confirming the western blot analysis results presented in Figure 3A and 3D. The level of MEK/ERK pathway inhibition shown in Figure 3A was also correlated with the reduction in the number of autophagic vacuoles as shown in Figures 3B and 3E.

The effect of BRAF and MEK inhibitors on cell viability and apoptosis in all three BRAFV600E colon cancer cell lines was examined next. In accordance to their effect on MEK pathway inhibition, presented in Figure 3A, PD0325901 was more efficient than PLX4720 in reducing cell viability in all three BRAFV600E colon cancer cell lines RKO, HT29 and Colo-205 after 24, 48 and 72 h treatments (Figure 3C). Treatment with MEK inhibitor PD0325901 had a similar effect on cell viability on all three BRAFV600E colon cancer cell lines, and the same was true for BRAF inhibitor PLX4720. On the other hand, treatment with MEK inhibitor resulted in the appearance of apoptotic markers, like cleaved Caspase-3 in HT29 (Sup. Figure 1, column 4, middle panel), and apoptotic Hoechst-stained nuclei in RKO (Figure 3C, upper right panel) while BRAF and MEK inhibitors did not cause any Caspase-3 cleavage in Colo-205 cells (Sup. Figure 2, columns 2 and 4 respectively, middle panel) under the same treatment conditions. Staining of RKO with LC3 antibody under confocal microscope revealed a scatterned staining pattern of this protein both in treated and untreated cells, though in a number of cells the staining was more compact and intense. Interestingly, treatment with BRAF and MEK inhibitors reduced the number of cells with high concentrated LC3 staining (Sup. Figure 3).

#### PI3K/MTOR inhibition

We initially examined the effect of PI-103, a dual PI3K/MTOR pathway inhibitor, on cell signalling and autophagy of BRAFV600E bearing colorectal cancer cell lines as shown in Figure 3A (lanes 4, 8, 12), Figure 3B (4^th^ column) and Figure 3E (3^rd^ column). Induction of autophagy was observed in the cell lines RKO and HT29 after treatment of 1μM PI-103 for 24 h. In RKO and HT29 cells, the increase in LC3II/LC3I protein level ratio was accompanied by a reduction in p62 protein levels, confirming a further activation of the autophagic machinery caused by PI-103 treatment (Figure 3D, lanes 3 and 9). Analysis of cell viability, autophagy and apoptosis after cell treatments with a larger number of PI3K/MTOR inhibitors on BRAFV600E cell lines was performed. Treatment with PI-103, a dual PI3K/MTOR inhibitor, significantly reduced cell viability in RKO (Figure 3C), while the number of MDC stained cells was increased by an average 20% (Figure 3E). On the other hand, treatments with AKT only inhibitor (GDC0941) or MTOR inhibitor (Rapamycin) reduced cell viability in all three treated mutant BRAF colon cancer cell lines in a similar way (Figure 3C). MDC staining in RKO cells after treatment with GDC0941 for 24 h did not change the number of MDC stained autophagic vacuoles (Figure 3E, 3^rd^ column, lower row), whereas the number of LC3 positive cells was increased (Sup. Figure 3). On the contrary, treatment of the same cell line RKO with Rapamycin resulted in a reduction in the number of cells showing highly concentrated LC3 staining (Sup. Figure 3), but the total number of MDC stained autophagic vacuoles did not change. Cell treatment with 1μM of the dual inhibitor PI3K/MTOR PI-103 for 24 h can trigger autophagic properties, as shown by the induced overexpression of LC3II protein levels, followed by a reduction in p62 protein levels in additional non BRAFV600E cancer cell lines Caco-2, DLD1 and HCT116 (Figure 3F). In Caco-2 and DLD1 cell lines, treatment with the dual PI3K/MTOR inhibitor PI-103 (Figure 3F, lanes 2, 6, 14), was necessary to increase LC3II protein levels and to activate autophagy, as was also confirmed by MDC staining of autophagosome vacuoles (Data not shown). Furthermore, LC3 staining pattern was altered due to PI-103 treatment in these cells (Figure 3G, 2^nd^ column, 3^rd^ row), even though DLD-1 control cells show high levels of LC3 stained aggregates (Figure 3G, 1^st^ coloumn, 3^rd^ row). On the contrary, inhibition of only one component of PI3K pathway using GDC0941 or Rapamycin was not sufficient to trigger these responses in the same cell lines, although in all Rapamycin-treated cell lines, p62 protein levels were significantly decreased (Figure 3F). Interestingly, in DLD-1 cells, treatment with GDC0941 or Rapamycin resulted in a significant reduction in highy-LC3 stained cells, confirming also the results of the WB, shown in Figure 3F. In HCT116 cells, none of the three inhibitors was able to drive LC3I to LC3II conversion in WB (Figure 3F), although PI-103 or Rapamycin treatment reduced the number of cells with highly concentrated LC3 staining (Data not shown). Levels of pAKT (ser473) and pS6R are shown to confirm inhibition of the related signalling pathway after treatment with PI3K/MTOR inhibitors (Figure 3F).

Taking the above under consideration, τhe MEK/ERK signalling pathway positively regulates autophagic markers and BRAF/MEK/ERK and PI3K/AKT/MTOR signalling pathways can differentially regulate the autophagic process.

### Individual autophagy inhibitors can differentially sensitize colon cancer cell lines towards apoptosis

#### 3-MA treatments induce partial response

**Figure 4:**
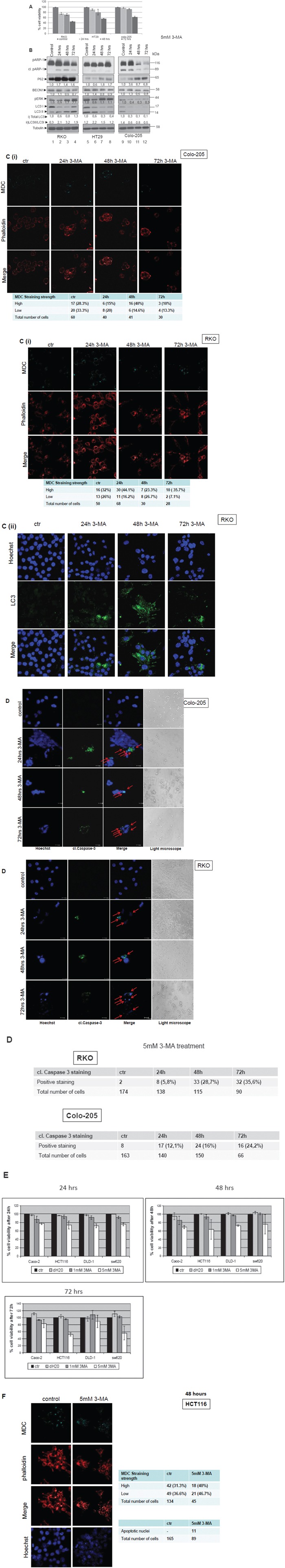
The role of autophagy in therapeutic response after treatment of colorectal cancer cell lines with the autophagic inhibitor 3-MA **A.** Cell Viability was measured, using the SRB assay, after treatment with 5mM of the autophagic inhibitor 3-MA [3-methyladenine (3-MA)] for 24, 48 and 72 h in BRAFV600E bearing cell lines RKO, HT29 and Colo-205. **B.** Western blot analysis after 3-MA treatment for 24 (lanes 2, 6, 10), 48 (lanes 3, 7, 11) and 72 (lanes 4, 8, 12) h: apoptotic cell death was tested by antibody against PARP (cleaved PARP), autophagy markers were evaluated by specific antibodies against LC3 I, II, BECN1 and p62. p-ERK levels were also evaluated by specific antibody. The quantification of LC3 reflects the whole protein levels as compared to the untreated sample in each cell line (i) and the ratio of LC3II/LC3I in each sample separately (ii). Protein levels were normalized against tubulin. **C.** The formation of autophagic vacuoles in RKO and Colo-205 cells due to treatments was determined with 0,1 mM of MDC (C(i), upper row, light blue) under confocal microscope. Cells were stained with phalloidin (C(i), middle row, red) in order to assess their number in the sample. In RKO, staining was also performed using Hoechst (4C(ii), upper row, blue), LC3 antibody (C(ii), middle row, green) and merged (C(ii), lower row). In both cases, the total number of cells and the number of stained cells from three different confocal images for each sample was recorded. **D.** Apoptotic marker staining under confocal microscopy for RKO and Colo-205 cells. Cells were incubated and stained with Hoechst (1^st^ column) and with cleaved Caspase 3 antibody (2^nd^ column) and merged (3^rd^ column) in order to detect the presence of apoptotic cell death under confocal microscopy, after treatment with 3-MA inhibitor for 24h (2^nd^ row), 48h (3^rd^ row) and 72h (4^th^), as compared to untreated control (1^st^ row). Cells were also visualized under optical microscope (4^th^ column). The total number of cells and the number of cells stained with cleaved Caspase 3 from three different confocal images for each treatment was recorded. **E.** The effect of 3-MA on cell viability was also examined in the BRAF wild type colon cancer cell lines Caco-2, HCT-116, DLD-1 and SW620. Cell Viability was measured, using the SRB assay, after treatment with 1 and 5mM of the autophagic inhibitor 3-MA [3-methyladenine (3-MA)] for 24 (upper left panel), 48 (upper right panel) and 72 h (lower panel). **F.** The formation of autophagic vacuoles in HCT116 cells due to the treatment with 5mM 3-MA inhibitor was determined with 0,1 mM of MDC (light blue staining, 1^st^ row) under confocal microscope. Cells were stained with phalloidin (middle row, red) in order to assess their number in the sample. Presence of apoptotic nuclei was detected by Hoechst staining (4^th^ row). The total number of cells and the number of stained cells from three different confocal images for each sample was recorded.

Since BRAFV600E cell lines presented remarkable autophagic properties, the efficiency of treatments with autophagy inhibitors was tested in these cell lines. Using the SRB-viability assay, cell responses of colorectal BRAFV600E bearing cancer cell lines (RKO, HT29 and Colo-205) were measured after treatments with the autophagy inhibitors 3-methyladenine (3-MA) (also a PI3KII inhibitor) and Bafilomycin A1 for 24, 48 and 72 h. The total number of cells was measured either by phalloidin or by Hoechst staining, as indicated. Due to inhibition of autophagy, cell viability was reduced by 10-20% after treatment with 5mM 3-MA in RKO, HT29 and Colo-205 cell lines for 24 and 48 h. Treatment of the three colon adenocarcinoma cell lines bearing BRAFV600E with 5mM 3-MA for 72 h in total, resulted in a 40-50% reduction in cell viability (Figure 4A). On the other hand, treatment of the same cell lines with 1mM 3-MA resulted in a maximum 15-20% reduction on their viability after 72 h (Sup. Figure 4A, upper panel). Treatment with 5mM 3-MA resulted in apoptotic cell death in all three BRAFV600E cell lines, as revealed by cleaved PARP, cleaved Caspase 3 and Hoechst staining under confocal microscopy. The presence of apoptotic cell death in RKO and Colo-205 cells after 3-MA treatment for 24 h (lanes 2 and 10, respectively), 48h (lanes 3 and 11, respectively) and 72h (lanes 4 and 12, respectively), was initially confirmed by the detection of PARP cleavage by Western blot analysis (Figure 4B). More evidence towards this direction was provided by Hoechst staining (Figure 4D, 1^st^ columns) and cleaved Caspase 3 (Figure 4D, 2^nd^ columns) by confocal microscopy: Upon 3-MA treatment, RKO and Colo-205 cells became sensitive to apoptotic cell death within the first 24 h (Figure 4D, RKO, 2^nd^ row) and (Figure 4D, Colo-205, 2nd row), while the number of cleaved Caspase 3-stained nuclei was increased by 35% and 24% respectively after 72 h of treatment. HT29 cells showed a higher resistance and exhibited signs of apoptotic nuclei after 48 h of 3-MA treatment (Data not shown). Evaluation of the effect of 3-MA treatment on autophagy markers was also performed (Figure 4B and 4C). Interestingly, expression levels of autophagy markers BECN1 and LC3 after 3-MA treatment were only reduced in Colo-205 cells (Figure 4B, lanes 10, 11, 12). Interestingly, the ratio between LC3 II and LC3 I, representing an autophagy marker, was increased upon longer 3-MA treatments in RKO (Figure 4B, lanes 2, 3, 4) cells, confirming a stabilization of autophagic marker expression upon treatment of cells with autophagy inhibitors [43, 52, 57]. Changes in total LC3 levels reflect the effect of 3-MA treatment on LC3 expression, while the increase in LC3II/I ratio represents activation of autophagy. The stabilization of LC3 in RKO cell line was also obvious after LC3 staining under confocal microscope, where LC3 aggregates were accumulated in the cytosole (Figure 4C(ii)). Furthermore, autophagy inhibitor 3-MA stabilizes protein levels of LC3 partner, p62, which is shown by a significant increase in p62 levels upon longer time treatments in all three cell lines (Figure 4B). Levels of pERK were also higher upon longer time points of 3-MA treatment, along with an increase in the LC3 II/I ratio, in these two cell lines RKO and HT29, indirectly indicating again a positive correlation between the BRAF/MEK/ERK proliferation pathway and the presence of autophagy (Figure 4B). On the other hand, the ratio between LC3 II and LC3 I, as well as pERK levels, was reduced in Colo-205 cells upon longer time 3-MA treatments. In accordance to this, autophagic vacuole formation was inhibited in RKO and Colo-205 cells after 72 h of 3-MA treatment, as confirmed by MDC staining upon confocal microscopy, while at the same time, cell population was reduced in both cell lines (Figure 4C(i)).

The effect of 3-MA treatment on cell viability, autophagy and apoptosis was also examined in the non-BRAFV600E colon cancer cell lines Caco-2, HCT116, DLD-1 and SW620. After treatment with 5mM 3-MA for 24, 48 and 72h, cell viability was reduced up to more than 40% in HCT116 and SW620 cell lines in 72h treatment (Figure 4E, lower panel), a result similar to that observed in BRAFV600E cell lines. On the other hand, Caco-2 and DLD-1 cell lines were more resistant to 3-MA treatments (Figure 4E). In the sensitive to 3-MA HCT116 cell line, bearing wtBRAF, the percentage of MDC stained vacuoles was increased upon 48h treatment, but the number of survived cells was significantly lower (Figure 4F, 2^nd^ column, lower row). The number of apoptotic nuclei was increased upon treatment, as revealed by Hoechst staining (Figure 4F, 4^th^ lane).

#### Bafilomycin A1 treatments efficiently sensitize tumor cells to apoptosis

**Figure 5:**
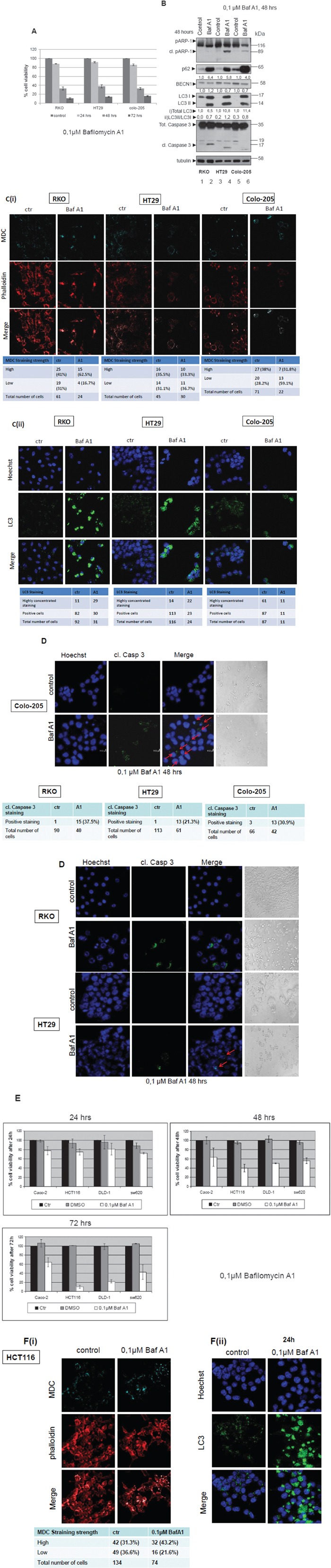
Autophagy inhibitor Bafilomycin A1efficiently sensitizes colorectal adenocarcinoma cells to apoptosis **A.** Cell Viability was measured, using the SRB assay, after treatment with 0.1μM of the autophagy inhibitor Bafilomycin A1 for 24, 48 and 72 h in BRAFV600E bearing cell lines RKO, HT29 and Colo-205. **B.** Western blot analysis after Bafilomycin A1 treatment for 48 h: apoptotic cell death was tested by specific antibodies against PARP (cleaved PARP) and Caspase-3 (cleaved Caspase-3), autophagy markers were evaluated by specific antibodies against LC3, BECN1 and p62. The quantification of LC3 reflects the whole protein levels as compared to the untreated sample in each cell line (i) and the ratio of LC3II/LC3I in each sample separately (ii). Protein levels were normalized against tubulin. **C.** The formation of autophagic vacuoles in RKO, HT29 and Colo-205 cells due to treatments was determined with 0,1 mM of MDC (C(i), upper row, light blue) under confocal microscope. In order to assess the total number of cells in the sample, cells were stained with phalloidin (C(i), middle row, red). Staining was also performed using Hoechst. (C(ii), upper row, blue), LC3 antibody (C(ii), middle row, green) and merged (C(ii), lower row). In both cases, the total number of cells and the number of stained cells from three different confocal images for each sample was recorded. **D.** Apoptotic marker staining under confocal microscopy for RKO, HT29 and Colo-205 cells. Cells were incubated and stained with Hoechst (1^st^ column) and with cleaved Caspase 3 antibody (2^nd^ column) and merged (3^rd^ column), in order to detect the presence of apoptotic cell death under confocal microscopy after treatment with Bafilomycin A1 inhibitor for 48 h, as compared to untreated control. Cells were also visualized under light microscope (4^th^ column). The total number of cells and the number of cleaved Caspase 3-stained cells from three different confocal images for each sample was recorded. **E.** The effect of 0,1 μM Bafilomycin A1 on cell viability was also examined in the BRAF wild type colon cancer cell lines Caco-2, HCT-116, DLD-1 and SW620. Cell Viability was measured, using the SRB assay, after treatment with 0,1μM of the autophagic inhibitor Bafilomycin A1 for 24 (upper left panel), 48 (upper right panel) and 72 h (lower panel). **F.** The formation of autophagic vacuoles in HCT116 cells due to the treatment with 0,1μM Bafilomycin A1 inhibitor was determined with 0,1 mM of MDC (F(i), light blue staining, 1^st^ row) under confocal microscope. Cells were stained with phalloidin (F(i), middle row, red) in order to assess their number in the sample. The total number of cells and the number of stained cells from three different confocal images for each sample was recorded. Staining was also performed using Hoechst (F(ii), upper row, blue), LC3 antibody (F(ii), middle row, green) and merged (F(ii), lower row).

Treatment of the BRAFV600E colon cancer cell lines with 0,1 μM Bafilomycin A1 (BafA1), another autophagy inhibitor, had an even stronger effect than that of 3-MA treatment on cell viability: cell viability was reduced to around 50% in all three cell lines, RKO, HT29 and Colo-205 after 48 h treatment, which was further reduced to 20% after 72 h treatment with BafA1 (Figure 5A). Treatment with a higher concentration of 1μM BafA1 resulted in a similar reduction of cell viability as compared to 0,1μM BafA1 in all three BRAFV600E cell lines tested (Sup. Figure 4A, lower panel) and Sup. Figure 4B therefore treatments with the lower concentration of 0,1μM BafA1 were selected for the next steps of the study.

Presence of apoptotic cell death was detected within the first 48 h of treatment, both by PARP and Caspase-3 cleavage after Western blot analysis (Figure 5B, lanes 2, 4, 6) accompanied by Hoechst (Figure 5D, 1^st^ column) and cleaved Caspase 3 (Figure 5D, 2^nd^ column) staining under confocal microscopy. Upon BafA1 treatment, RKO, HT29 and Colo-205 cells became sensitive to apoptotic cell death, within the first 24 h (Figure 5D, RKO, 2^nd^ row), (Figure 5D, HT29, 2^nd^ row), (Figure 5D, Colo-205, 3^rd^ row). Evaluation of the effect of 3-MA treatment on autophagy markers was also performed. Also in this case of BafA1 treatment, there was a controversial increase on LC3II levels, as well as on the LC3 II/ LC3I ratio upon treatment with BafA1 for 48 h in all BRAFV600E mutant cell lines (Figure 5B, lanes 2, 4, 6), confirming a stabilization of autophagic marker expression upon treatment of cells with autophagy inhibitors. The changes in total LC3 levels reflect the effect of BafA1 treatment on LC3 expression, while the increase in LC3II/I ratio represents the activation of autophagy. p62 protein levels were also remarkably increased upon treatment, following LC3 stabilization (Figure 5B). BECN1, the other autophagic marker was upregulated in RKO cells after BafA1 treatment (Figure 5B, lane 2), but was downregulated in HT29 (Figure 5B, lane 4) and Colo-205 (Figure 5B, lane 6), in which PARP cleavage is more obvious. In accordance to this, the percentage of autophagic vacuoles was higher by around 8% in RKO and by 24% in Colo-205 cells after 48 h of Bafilomycin A1 treatment, as confirmed by MDC staining upon confocal microscopy, while at the same time, cell numbers were reduced in all three cell lines (Figure 5C(i)). Moreover, staining with LC3 revealed an accumulation of protein aggregates in all three cell lines, RKO, HT29 and Colo-205, revealing the protein level stabilization due to treatment with an autophagy inhibitor (Figure 5C(ii)).

The effect of BafA1 treatment on cell viability, autophagy and apoptosis was also examined in the non-BRAFV600E colon cancer cell lines, Caco-2, HCT116, DLD-1 and SW620. Upon treatment with 0,1μM BafA1, cell viability was reduced by more than 80% in HCT116 and DLD-1 cell lines after 72 h treatment (Figure 5E, lower panel), a result similar to that in BRAFV600E cells. On the other hand, Caco-2 intermediate adenoma cells were more resistant to BafA1 action by a difference around 40% in cell viability after 72 h treatment, when compared to all the examined adenocarcinoma cell lines (Figure 5E, lower panel). Furthermore, in HCT116, MDC staining of autophagic vacuoles did not significantly change, (Figure 5F(i)). Despite the fact that the total number of cells was reduced after 24h treatment, no indication of apoptotic nuclei was detected under these conditions (Figure 5F(ii)). Staining with LC3 revealed an accumulation of protein aggregates, revealing also here the protein stabilization due to treatment with an autophagy inhibitor (Figure 5F(ii)).

Taking the above under consideration, it can be proposed that autophagy presents a cytoprotective role in colorectal cancer cell lines, since inhibition of autophagy by two established autophagy inhibitors is remarkably correlated with decrease in cell viability and appearance of apoptosis. Remarkably, autophagy inhibitor BafA1 appears to be a very potent anti-cancer agent in the examined colorectal adenocarcinoma cell lines, suggesting to its further exploitation.

### Inhibition of autophagy by 3-MA can sensitize resistant BRAFV600E bearing colorectal cancer cell lines to the specific BRAFV600E inhibitor PLX4720 towards apoptotic cell death in a synergistic manner

BRAFV600E bearing colorectal adenocarcinomas are resistant to current treatments, even to Vemurafenib (PLX4032), a targeted BRAFV600E drug. Thus, studies have been focusing on combinatorial drug treatments, in an effort to overcome tumor resistance. Here, the anti-cancer efficiency of autophagy inhibitors was tested, on rational combinatorial treatments with PLX4720, a BRAFV600E inhibitor- Vemurafenib analog and lead compound, in an effort to propose new effective combinatorial treatments to sensitise resistant BRAFV600E mutant colorectal cancers to BRAFV600E inhibitors.

**Figure 6:**
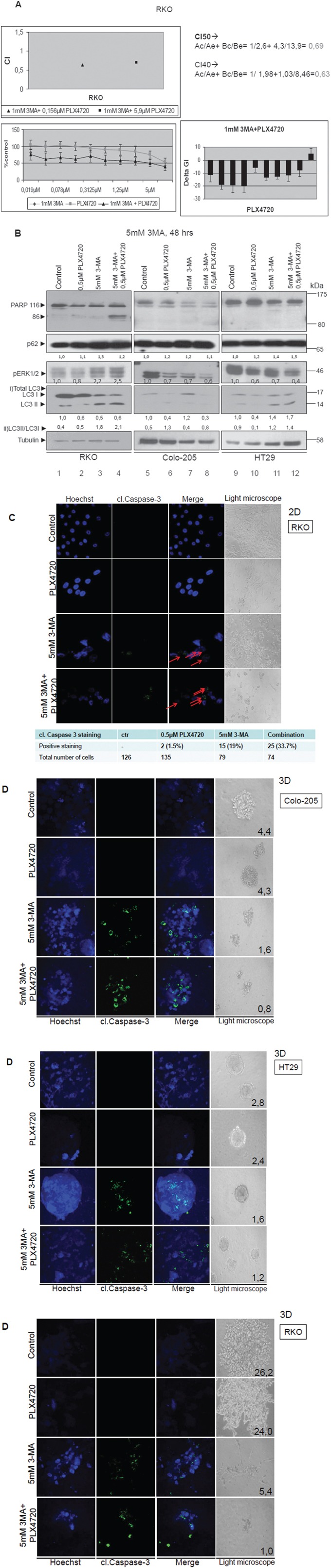
Autophagy-mediated resistance mechanism can be overcome by synergistic treatment of PLX4720 with 3-MA in BRAFV600E cells **A.** Cell viability of the BRAFV600E bearing cell line RKO after mono- and pre-treatment with autophagic and BRAF inhibitors. RKO cells were treated with either 3-MA (GI_50_=2,6 mM) or PLX4720 (GI_50_=13,9μM) or with their combination for 48 h. Upper left panel: Combination Indices are shown, CI<1 synergism, CI=1 additivity and CI>1 antagonism, with the values shown in the upper right panel, N=4, bars are standard errors. Lower left panel: The effect of PLX4720 with or without a fixed dose (1mM) of 3-MA on cell viability is shown. Lower right panel: Additive, synergistic or antagonistic effects are shown: values above zero indicate antagonism, equal to zero additivity and below zero synergism. The strongest synergistic effect is delivered by the combination of 1mM 3-MA with 0,156μM PLX4720. **B.** Western blot analysis of PARP, pERK1/2, LC3B, p62 and tubulin protein levels in RKO (lanes 1-4), Colo-205 (lanes 5-8) and HT29 (lanes 9-12) cell lines after treatment with either 0,5μM PLX4720 (lanes 2,6,10) or 5mM 3-MA (lanes 3, 7,11) or their combination (lanes 4,8,12). For the combination treatments, cells were pre-treated for 2 h with 5mM of the autophagic inhibitor 3-MA and then co- treated with 5mM 3-MA and 0,5μM PLX4720 for 48 h in total. The quantification of LC3 reflects the whole protein levels as compared to the untreated sample in each cell line (i) and the ratio of LC3II/LC3I in each sample(ii) **C.** Light and confocal microscope images of two-dimensional culture in RKO cell line after treatment with either 0,5μM PLX4720 (2^nd^ row) or 5mM 3-MA (3^rd^ row) for 48 h as compared to control untreated cells (1^st^ row). For the combination treatments, cells were first incubated for 2 h with 5mM of the autophagic inhibitor 3-MA and then co- treated with 5mM 3-MA and 0,5μM PLX4720 for 48 h in total (4^th^ row). For apoptotic marker staining under confocal microscopy, RKO cells were incubated and stained with Hoechst (1^st^ column) and with cleaved Caspase 3 antibody (2^nd^ column) and merged (3^rd^ column) in order to detect the presence of apoptotic cell death, as compared to untreated control (1^st^ row). The total number of cells and the number of cleaved. Caspase 3-stained cells from three different confocal images for each sample was recorded. Cells were also visualized under light microscope (4^th^ column). **D.** Light and confocal microscope images of three-dimensional culture in RKO, HT29 and Colo-205 cell lines after treatment with either 0,5μM PLX4720 (2^nd^ row) or 5mM 3-MA (3^rd^ row) for 48 h as compared to control untreated tumors (1^st^ row). For the combination treatments, tumor cells were first incubated for 2 hr with 5mM of the autophagic inhibitor 3-MA and then co- treated with 5mM 3-MA and 0,5μM PLX4720 for 48 h in total (4^th^ row). Tumor treatments started after twelve-day tumor formation. For apoptotic marker staining under confocal microscopy, RKO tumors were incubated and stained with Hoechst (1^st^ column) and with cleaved Caspase 3 antibody (2^nd^ column) and merged (3^rd^ column) in order to detect the presence of apoptotic cell death, as compared to untreated control (1^st^ row). Tumors were also visualized under light microscope and their mass was measured (4^th^ column).

Pre-treatment of BRAFV600E colon carcinoma cells with autophagy inhibitor 3-MA followed by PLX4720 and 3-MA combinational treatment, resulted in a synergistic effect on colorectal cancer cell viability. This was accompanied by appearance of apoptotic properties in 2- and 3- Dimensional cultures:

In detail, BRAFV600E colorectal cancer cell line RKO was treated separately with increasing concentrations of the autophagy inhibitor 3-MA (GI_50_=2,6 mM) (Data not shown) and the specific BRAFV600E inhibitor PLX4720 (GI_50_=13,9μM) (Figure 6A, lower left panel), as well as with a combination of increasing concentrations of PLX4720 with a fixed dose of 1mM 3-MA for 48 h (Figure 6A, lower left panel) in order to assess the effect on cell viability. The fixed concentration of 1mM 3-MA was selected as the concentration where the inhibitor alone had a modest effect GI on cell viability. CIs (Combination Index) were calculated for the combination of different PLX4720 concentrations with 1mM of 3-MA [37–39] (CI<1 synergism, CI=1 additivity and CI>1 antagonism, N=4, bars are standard errors). The analysis demonstrated synergism, since CI_40_=0,63 and CI_50_=0,69 were both <1 (Figure 6A, upper left and right panel). For further confirmation, the synergistic effect of the two inhibitors was also estimated using the equation DeltaGI=GI_PLX4720_+GI_3-MA_-GI_combination_, based on the Bliss Independence Model. Additive, synergistic or antagonistic effects are shown in the lower right panel: values above zero indicate antagonism, equal to zero additivity and below zero synergism. The analysis has shown below zero values in all but two combinatorial points, thus demonstrating synergism for the combination of 3-MA with specific concentrations of PLX4720 (Figure 6A, lower right panel). As presented in this graph (Figure 6A, lower right panel), there is a PLX4720 dose dependent pattern for synergism, in all but the lowest or highest PLX4720 concentrations. Notably, the best synergistic effect on cell viability was delivered by combination of 1mM 3-MA with 0,156μM PLX4720.

In addition, pre- treatment of BRAFV600E colon carcinoma cells with 5mM 3-MA followed by 0,5μM PLX4720 treatment, resulted in a decrease of RKO cell viability by 60% and around 40% in Colo-205 and HT29 cells. Remarkably, the protocol which is based on pre- treatment of cells with 3-MA, followed by combinatorial treatments of 3-MA with PLX-4720 resulted in a 16% synergistic effect in the BRAFV600E adenocarcinoma cell line RKO, when 5mM of 3-MA was used (Data not shown).

The effects of efficient drug combinatorial and synergistic treatments on cell death properties were further analysed. Therefore, protein levels of PARP, P-ERK1/2, LC3, p62 and tubulin were compared after treatment of RKO, HT29 and Colo-205 cells with PLX4720 or 3-MA individual treatments, pre-treatment with 3-MA and then combination treatments of 3-MA with PLX4720. Pre-treatment of cells with 3-MA followed by co-treatments of 3-MA with PLX4720 showed clear evidence of apoptosis, confirmed by detection of cleaved PARP using western blot analysis in RKO cells (Figure 6B, lane 4). These results were validated with Hoecsht staining (Figure 6C, 1^st^ column, 4^th^ row), caspase-3 (Figure 6C, 2^nd^ column, 4th row) cleavage detection and their merged staining (Figure 6C, 3^rd^ column, 4^th^ row) as compared to the control untreated cells (Figure 6C, 3^rd^ column, 1^st^ row) in 2D cell culture. In RKO cell line, Caspase 3-stained cells were higher by 14% after combinatorial treatment with 3-MA and PLX4720, compared to 3-MA mono-treatment. Furthermore, the ratio between LC3II and LC3I was higher after combination treatment with PLX4720 and 3-MA and the same was true for p62 protein levels, revealing a stabilization of the protein levels of these two autophagy markers, due to the treatment (Figure 6B).

To test treatment conditions that mimic the real tumor microenvironment, cells were grown in 3D cultures and formed tumors in matrigel. Apoptotic nuclei were also detected and quantified using the same combinatorial drug protocol in 3D cell cultures of all three BRAFV600E cell lines tested, as observed by Hoechst staining and cleaved Caspase 3 antibody under confocal microscopy. Furthermore, the tumor mass is significantly reduced in 3D culture, where cells appear apoptotic in pre- treatment of tumors with 3-MA followed by co-treatment of 3-MA with PLX4720. Interestingly, in contrast to the 2D culture, the apoptotic effect of 3-MA and PLX4720 combination is more evident and strong in the 3D culture in all of the examined BRAFV600E colorectal cancer cell lines (Figure 6D, RKO, HT29, Colo-205). As already demonstrated in previous figures, treatments involving autophagy inhibitors generally stabilize the expression of autophagic markers, like LC3 (Figure 6D, lanes 3-4, 7-8, 11-12). The changes in total LC3 levels reflect the treatment results of the individual inhibitors and their combination on LC3 expression, while the increase in LC3II/I ratio represents the activation of autophagy.

Combination treatments with PLX4720 were also performed for BafA1, in order to examine their potential synergistic effect on BRAFV600E colorectal cancer cell line viability and cell death, as tested by SRB assay and confocal microscopy for Hoechst/Caspase-3, respectively, but no evident synergy was observed as shown in Sup. Figure 5.

This data indicate autophagy as a putative resistance mechanism of colon cancer cell lines bearing mutant BRAFV600E to BRAFV600E inhibitor PLX4720, since inhibition of autophagy by 3-MA could sensitize the cells to PLX4720 and apoptosis, thus indicating a promising new combination for BRAFV600E colorectal tumor treatment to be further exploited *in vivo*.

## DISCUSSION

The present study investigates the association of mutant KRAS, BRAF (and PIK3CA) in advanced and recurrent autophagic process through regulation of the two autophagic markers BECN1 and especially LC3 in a number of CRC cell lines. The association of *BRAF* mutant genes with increased autophagic activity was exploited towards establishing novel as well as efficient anti-cancer treatment protocols, which involve either inhibitors of autophagy as a single agent treatment or their rational combination with BRAF600E targeting drugs.

### BRAFV600E induces autophagy in colorectal tumor cells

Autophagosome formation requires LC3, a protein conjugation system that resembles ubiquitin and is important for transport and maturation of the autophagosome [45]. To our knowledge, this is the first study to demonstrate that high expression of the autophagic factor LC3 is stimulated by the *BRAF* oncogene as a notable example of an oncogenic kinase regulating autophagy and to indicate the cytoprotective role of autophagy in colorectal cancer cell lines bearing BRAFV600E [47]. Furthermore, an association between EMT and autophagy has already been revealed in many studies, where EMT markers are co-expressed with the autophagic marker LC3 [46]. In the present study we also noticed a relevant correlation between cell lines with EMT characteristics and a low ratio of LC3II/ LC3I protein expression.

BRAFV600E is a major tumorigenic oncogene. The presented data show that BRAFV600E can induce autophagy through over-activation of MEK/ERK pathway. This pathway not only can induce the expression of LC3, but can also trigger the autophagosome formation in BRAFV600E colon cancer cell lines. Furthermore, it is shown here that the two signaling pathways MEK/ERK and AKT/MTOR can differentially regulate the autophagic process: the MEK/ERK pathway is able to increase the activity of basic autophagy and related cell properties, and BRAF/MEK inhibitors can reduce the expression of autophagic markers. The AKT/MTOR pathways inhibit autophagic mechanisms and properties. Notably, simultaneous inhibition of AKT and MTOR pathways resulted in a remarkable induction of autophagic markers and related cell properties-morphology in the majority of the treated cell lines. Many studies have demonstrated that inhibition of MEK/ERK and AKT/MTOR pathways can lead to cell death [48, 49]. Autophagy is known to play an important role in tumor cell survival, while in cancer cells, autophagy is used as a way to deal with cellular stress [50]. Once autophagy-related genes were inhibited, cell death was potentiated in several cases [51]. Furthermore, inhibition of autophagy has also been shown to enhance the effectiveness of anticancer therapies.

Mutant *KRAS* oncogene can decrease the expression of the autophagic marker BECN1. Nevertheless, the ratio between LC3II and LC3I protein levels increases in the presence of *KRAS* oncogene, which further results in p62 downregulation, indicating an induction of the autophagic process, as shown in the current study utilizing colorectal cancer cells. When mutant KRAS co-exists with mutant PIK3CA in the same tumor, the protein levels of autophagic factor LC3 are recorded high. While mutant KRAS is connected with upregulation of autophagic markers and autophagy, several other studies support the opposite hypothesis [10, 52]. We report here that expression of *HRASV12* oncogene, an isoform of the *RAS* family, does not affect the autophagy state, though enhancing the levels of the autophagic marker BECN1, which has been described to increase basal autophagy and limit the starvation-induced autophagy [10–13]. As already mentioned, BRAFV600E oncogene increases significantly the level of LC3, thus inducing autophagic markers. Therefore this study provides for the first time, a comparative study of *BRAF*, *KRAS* and *HRAS* oncogenes regarding their differential regulation of autophagic markers and related properties in colorectal tumor cells.

BECN1 and LC3 were downregulated in the majority of examined tumors. In a previous study [53] it was shown that in 56% of breast cancers there was a significant decrease of BECN1 protein expression in cancer cells compared to normal breast epithelial cells. In both colorectal and gastric cancers where BECN1 mutations rarely occur, decrease of BECN1 expression in cancer cells could be an inactivation mechanism of BECN1 tumor suppressor functions. Another study by Giatromanolaki A et al has found that perinuclear LC3A accumulation in colorectal tumor cells is a marker of good prognosis, presumably reflecting a basal autophagic activity [54]. Here, we provide further evidence for the strong association of BRAFV600E, another mutant oncoprotein of RAS pathway, with increased expression of autophagic markers in colorectal tumors and cancer cell lines.

### Rational efficient combinatorial treatment protocols against resistant BRAFV600E colorectal tumors, utilizing BRAFV600E and autophagy inhibitors

Autophagy-related stress tolerance can enable cell survival by maintaining energy production that can lead to tumor growth and anti-cancer therapy resistance [55]. Treatment of cells that have acquired therapeutic resistance with autophagy inhibitors shows particular promise in many preliminary studies [56]. When bafilomycin A1 was used to inhibit γ-irradiation-induced autophagy in breast, prostate and colon cancer tumors, cells underwent apoptosis, and the antitumor effect of γ-irradiation was increased. These results indicate that autophagy could be a defense mechanism of cancer cells against radiation-induced apoptosis. So if autophagy protects cells from drug-induced apoptosis, agents that block autophagy might enhance the antitumor efficacy of cytotoxic agents [57, 58]. Fundamental knowledge of the role of autophagy in the immune system may provide new clues to improve immunotherapy of cancer in many cases. Highlights of ongoing phase I and phase II clinical trials for first-generation inhibitor of autophagy, hydroxy-chloroquine, rise the potential for clinical application in this field [22].

In the current study, inhibition of autophagy either by 3-MA (inhibition in early stage of autophagophore formation) or Bafilomycin A1 (BafA1) (inhibition in later stage- in maturation of autophagophore) leads to a strong reduction in cell viability and activation of apoptosis, in cells treated only with BafA1. Mono-treatments with BafA1 were very efficient in all colorectal adenocarcinoma cell lines tested, but not in the intermediate adenoma cell line Caco-2. It is proposed that further studies are necessary for its establishment as an efficient therapeutic agent. Treatment of colorectal adenocarcinoma cells with 5mM 3-MA provides evidence that the most resistant cell lines DLD-1 and HT29 present high levels of autophagic markers like LC3II/LC3I ratio and MDC staining. On the other hand, the partial response of colorectal cancer cells to 3-MA has been exploited here towards its potential synergistic action with targeted therapeutics.

The specific inhibitor PLX4032 (Vemurafenib) has given impressive results in clinical trials involving patients with BRAFV600E melanoma, causing significant tumor regression [59, 60]. In melanoma, combinatorial treatments of BRAFV600E targeting drugs with novel autophagy inhibitors may overcome resistance [61]. Colorectal cancer and other cancer types show intrinsic resistance to BRAFV600E specific inhibitors. Combinatorial treatments that include BRAF and EGFR inhibitors achieve encouraging preclinical, as well as clinical results [26, 62]. Other studies support that Akt, a member of the PI3K/Akt/MTOR pathway, could also drive tumor resistance to BRAFV600E inhibitors and thus simultaneous inhibition of those two pathways (BRAF and PI3K) is needed [33, 63, 64].

Inhibition of autophagy not only can contribute to decreased tumor cell numbers, but will also sensitize cancer cells to kinase inhibitors, like vemurafenib, to trigger the apoptotic cell death [50, 53]. In a recent study in a brain tumor bearing BRAFV00E, the effect of chemotherapeutic treatment using vemurafenib following autophagy inhibition by chloroquine was enhanced [65, 66]. The effect of another anti-cancer drug, pouranole, is increased after the combination with the specific autophagic inhibitor 3-MA and results in tumor cell death through apoptosis [67]. In addition, Bokobza et al, 2014, have shown the reduction of a tumor right after autophagy inhibition by chloroquine in combination with an AKT kinase inhibitor [68]. In our study, solid evidence presenting the protective action of autophagy in colon cancer cells bearing BRAFV600E is provided. Data presented here provide strong evidence that pre-incubation with 3-MA followed by concomitant treatment with BRAFV600E targeting PLX4720 inhibitor leads to a synergistic effect that sensitizes cells to apoptotic cell death. [40, 41]. Therefore, BRAFV600E induced over-expression of key autophagy molecules and markers presented in the first part of this study, has been further exploited by providing solid evidence of efficient combinatorial treatments of anti-BRAFV600E and autophagy inhibitors, that can sensitize resistant BRAFV600E colorectal tumor cells to apoptosis *in vitro*. These combinatorial treatments involving a BRAFV600E specific inhibitor and an autophagy inhibitor could be further exploited for their potent efficiency *in vivo*. Unlike melanomas, where the targeted BRAFV600E inhibitor vemurafenib leads cells to death through apoptosis [59], it is likely that the observed resistance of colon cancer cells and tumors to vemurafenib is partially due to the protective effect of autophagy in these cells.

In conclusion, it is clear from this *ex vivo* study that autophagy is triggered by BRAFV600E and the signalling pathway RAF/MEK/ERK. Furthermore, the cytoprotective role of autophagy in colon cancer cell lines bearing this mutation is a new and promising target for the future anti- cancer strategy on patients with mutant BRAFV600E. It is also proposed that autophagy could be targeted in nonBRAFV600E colorectal tumors by selected existing or novel autophagy inhibitors.

## MATERIALS AND METHODS

### Cell lines

DLD-1, HCT116, RKO, HT29, Colo-205 and SW620 human colon adenocarcinoma and Caco-2 colon intermediate adenoma cell lines were obtained from American Type Culture Collection (ATCC) and DKO-4 and HKE-3 were kindly provided by S. Shirasawa et al [35]. Oncogenic cell models used in the present study were generated in Caco-2 cells using stable transfection in order to constitutively express HRASG12V (Caco-H), KRASG12V (Caco-K) [36] or BRAFV600E (Caco-BR) oncogenes [37]. Clones RKOshBR 2 and RKOshBR 10 were derived after silencing of BRAFV600E with stable transfection of shRNA against BRAFV600E in RKO cells. The small inhibitory duplex shRNA oligo was cloned into the HindIII and BglII sites in pSUPER (Oligoengine, Seattle, WA), as described in Makrodouli et al., 2011 [38]. The sense strand of the shRNA pSUPER BRAFV600E insert was BRAFmutshRNA:gatccccGCTACAGAGAAATCTCGAT-ttcaagagaATCGAGATTTCTCTG TAGCtttttggaaa [39]. RKO cells were also transfected with the empty vector (pSUPER using the CaPO4 precipitation technique and selected with Geneticin (Invitrogen). The names of clones used in this study are: RKOpSUPER-1 (for empty vector) and RKOshBR 2 and 10 cell clones (for BRAFV600E silencing). All cell lines used in this study were grown in D-MEM medium supplemented with 10% FBS, antibiotics and amino acids (all from Invitrogen).

### Western blotting

Whole cell lysates were prepared with lysis buffer. Extracts were resolved on SDS-PAGE, and transferred to nitrocellulose membrane (Whatman, Scheicher & Schuell, Dassel, Germany). Membranes were incubated with the specific antibodies overnight at 4°C, washed and incubated with the appropriate secondary antibody, for 1 h at room temperature. Antibodies were used against: pAKT (s473) #9271, pS6R (s235/236) #2211, pMEK (s217/221) #9121, LC3B(D11) #3868, pBRAF #2996, total Caspase-3 #9662, cleaved caspase-3 #9661 from Cell Signalling (Danvers, MA, USA), BECN1 (sc-10086), SQSTM1/p62 (sc-28359), PARP (sc-7150), pERK (sc-7583), and Tubulin (sc-8035) from Santa Cruz (Biotechnology, Inc. 2145 Delaware Avenue Santa Cruz, CA 95060 USA). Antibody signal was obtained with the enhanced chemiluminescence plus Western blotting detection system (Amersham Biosciences, Uppsala, Sweden) after exposure to Kodak Super RX film. Values were measured using the Image-Quant software (Amersham Biosciences) and protein levels were normalized against tubulin. Experiments were independently repeated three times and standard deviation is presented.

### Inhibitors

BRAF/MEK/ERK and AKT/MTOR signalling pathways were inhibited using specific kinase inhibitors. PI-103 #S1038 SELLECKCHEM, Rapamycin #R8781 SIGMA, GDC0941 #S1065 SELLECKCHEM, PD 0325901 #PZ0162 SIGMA, PLX4720 #S1152 SELLECKCHEM,, Autophagy inhibitors: 3-methyladenine (3-MA), #sc205596, Bafilomycin A1 #sc-201550 Santa Cruz Biotechnology, Inc.

### Two dimensional culture

For the 2D culture experiments, cells (5000 cells/well) were grown on coverslips in 24 well plates in medium, at 37°C. Photographs of the 2D cultures were taken under light and confocal microscope (Leica 626 TCS SPE confocal laser scanning microscope after the appropriate staining). LAS AF software was used for image 627 acquisition (Leica Lasertechnik, Heidelberg, Germany). For the confocal analysis, cells were labeled with 0.1 mM monodansylcadaverin (MDC) (Sigma) in PBS at 37°C for 15 min. Cells were fixed with 4% paraformaldehyde, washed with PBS and immediately analyzed in confocal to detect the autophagic vacuoles. MDC is an auto-fluorescent marker that preferentially accumulates in autophagic vacuoles after which treatment were applied for indicated incubation times. MDC accumulation in autophagic vacuoles is due to a combination of ion trapping and specific interactions with vacuole membrane lipids. Cell cytoskeleton was stained with phalloidin (Alexa Fluor 546, A22283, Life technologies). Nuclei were stained with Hoechst No. 33342 (Sigma, B2261) for apoptosis detection; Cleaved caspase-3, another marker of apoptosis, was detected by cleaved caspase-3 antibody.

### Three dimensional culture

For three-dimensional culture experiments, cells were grown in 24-well plates on 20% Matrigel (BD Bioscience) that was allowed to set for 15 minutes at 37°C in order to form a gel of 1 mm thickness. The bottom later was then covered with 2×104 cells mixed 1:1 with 4% Matrigel in a total volume of 600 μl. Growth medium containing 2% matrigel was replaced every 2 days and the cells were left to grow for 12–14 days to allow development of extensive tubule network, after which treatment were applied for indicated incubation times. Photographs of the three-dimensional cultures were taken using a Nikon Eclipse T-200 inverted phase-contrast microscope equipped with an Olympus digital camera. The nuclei were stained with Hoechst No. 33342. The cleaved caspase three was detected with cleaved caspase-3 specific antibody.

### Cell viability assay

For growth studies the sulforhodamine B (SRB, SIGMA) assay was used. Firstly, tumor cells were seeded into 96-well micro titer plates and were allowed to attach overnight. Thereafter, the cell number in treated versus control wells was estimated after treatment with 10% trichloroacetic acid and staining with 0.4% SRB in 1% acetic acid. The percentage of viable cell was plotted each time. SD was used for error bar generation.

The median effect analysis was used for calculation of combination indices (CI) [40, 41]. Synergism was determined using the method previously described [41, 42].

### RNA Extraction/reverse transcription and real time-PCR

Total RNA isolation from cultured cells as well as cancer specimens was performed using the Trizol reagent (Invitrogen, Karlsruhe, Germany). Reverse transcription was carried out from 3.0 μg of purified RNA using the SuperScript Reverse Transcriptase (Invitrogen, Karlsruhe, Germany) following the manufacturer's instructions. Real-time quantification at the mRNA level was carried out in 96-well PCR plates using a Bio-Rad iCycler and the iQ5 Multicolor real-Time PCR detection system (Bio-Rad, Hercules CA, USA). Each reaction contained 1× iQ SYBR Green Supermix (Bio-Rad, Hercules CA, USA) and 150 nmol/L of each primer. All genes were tested in duplicates. Results were analyzed on the iCycler software. Values were normalized to GAPDH. Primers used were the following: *GAPDH*: GAAGGTGAAGGTCGGAGT (Fw) and CATGGGTGGAATCATATTGGAA (Rv), *LC3*: GAGAAGCAGCTTCCTGTTCTGG (Fw) and GTGTGCGTTCACCAACAGGAAG (Rv). *BECN1*: GGCTGAGAGACTGGATCAGG (Fw) and CTGCGTCTGGGCATAACG (Rv).

Tumor specimens undergoing surgery for CRC at G. Genimatas General Hospital of Athens participated in the study and gave written consent. The study protocol was approved by the Medical Ethical Review Committee of G. Genimatas General Hospital of Athens. Upon surgery, resected samples were placed in 5 ml RNA*later* (Ambion, Austin, USA) to preserve RNA integrity and transferred to the laboratory.

## SUPPLEMENTARY FIGURES


