# Supplementary material for: BRAF associated autophagy exploitation: BRAF and autophagy inhibitors synergise to efficiently overcome resistance of BRAF mutant colorectal cancer cells
Source: Oncotarget. 2016 Jan 18;7(8):9188–221. doi: 10.18632/oncotarget.6942 (PMC4891035; doi:10.18632/oncotarget.6942)
Supplement: Supplementary file 1 [file oncotarget-07-9188-s001.pdf]

SUPPLEMENTARY FIGURES

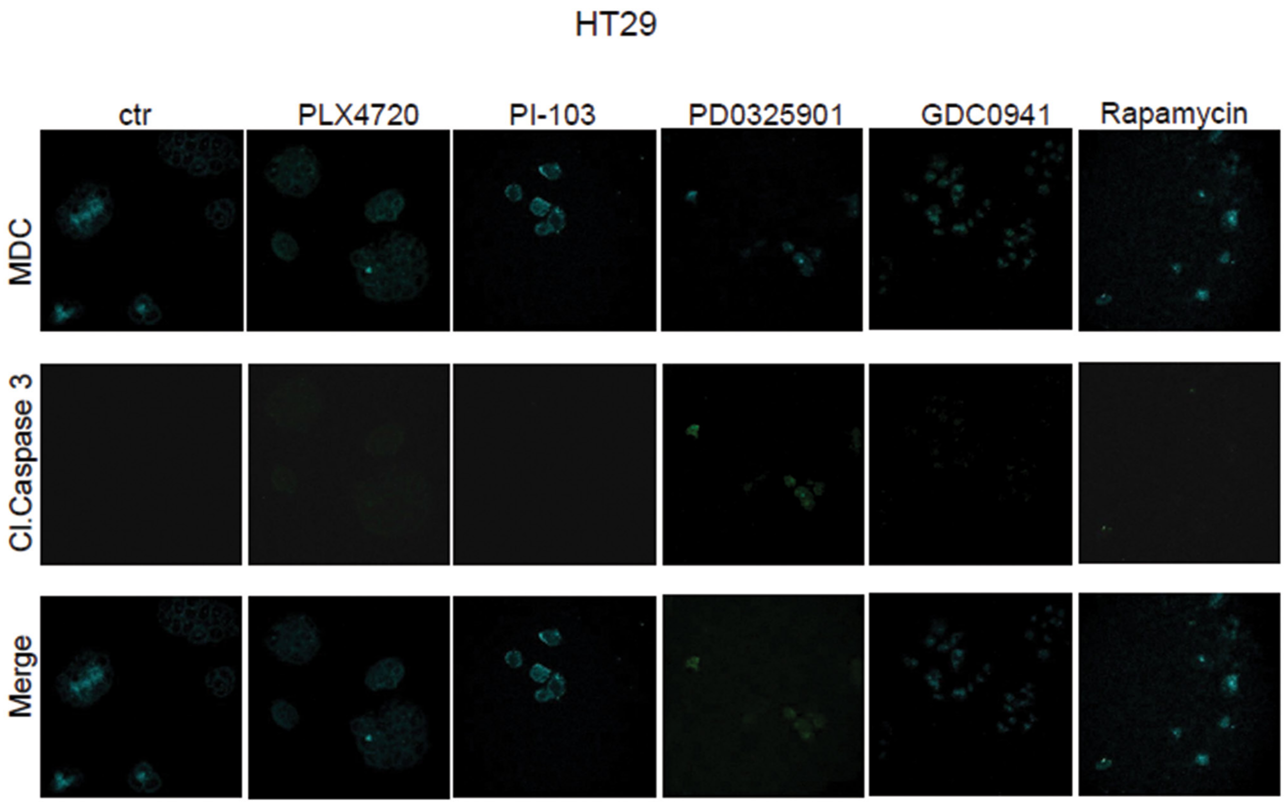

Supplementary Figure S1: Confocal microscope images of two-dimensional culture in HT29 cell line after treatment with 1µM of each of the following BRAF/MEK/ERK and PI3K/MTOR pathway inhibitors; PLX4720 (2nd column), PI-103 (3rd column), PD0325901(4th column), GDC0941(5th column), Rapamycin (6th column) as compared to control untreated cells (1st column). Cells were stained with either MDC (upper row) or cleaved Caspase-3 antibody (middle row), or merged staining (lower row), in order to detect autophagic vacuoles (MDC) and apoptotic cell death (cleaved Caspase-3).

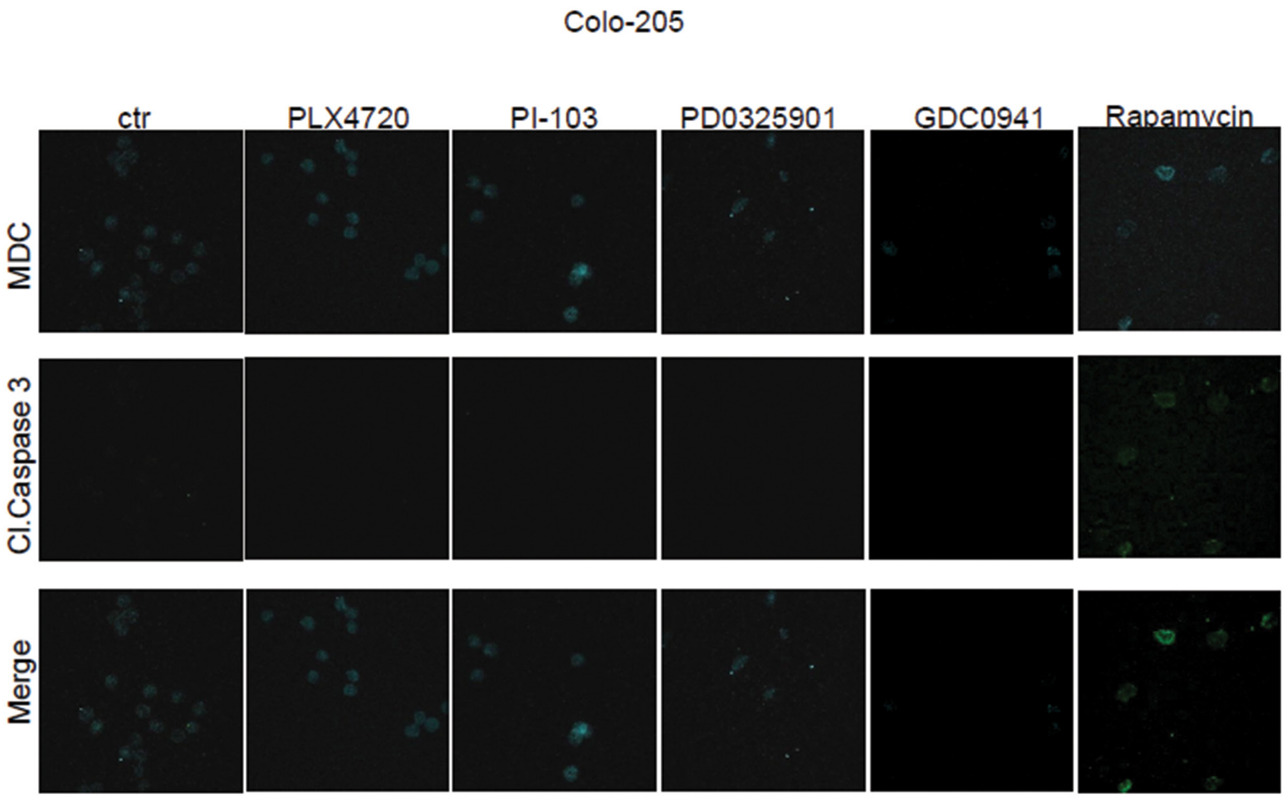

Supplementary Figure S2: Confocal microscope images of two-dimensional culture in Colo-205 cell line after treatment with 1µM of each of the following BRAF/MEK/ERK and PI3K/MTOR pathway inhibitors; PLX4720 (2nd column), PI-103 (3rd column), PD0325901(4th column), GDC0941(5th column), Rapamycin (6th column) as compared to control untreated cells (1st column). Cells were stained with either MDC (upper row) or cleaved Caspase-3 antibody (middle row), or merged staining (lower row), in order to detect autophagic vacuoles (MDC) and apoptotic cell death (cleaved Caspase-3).

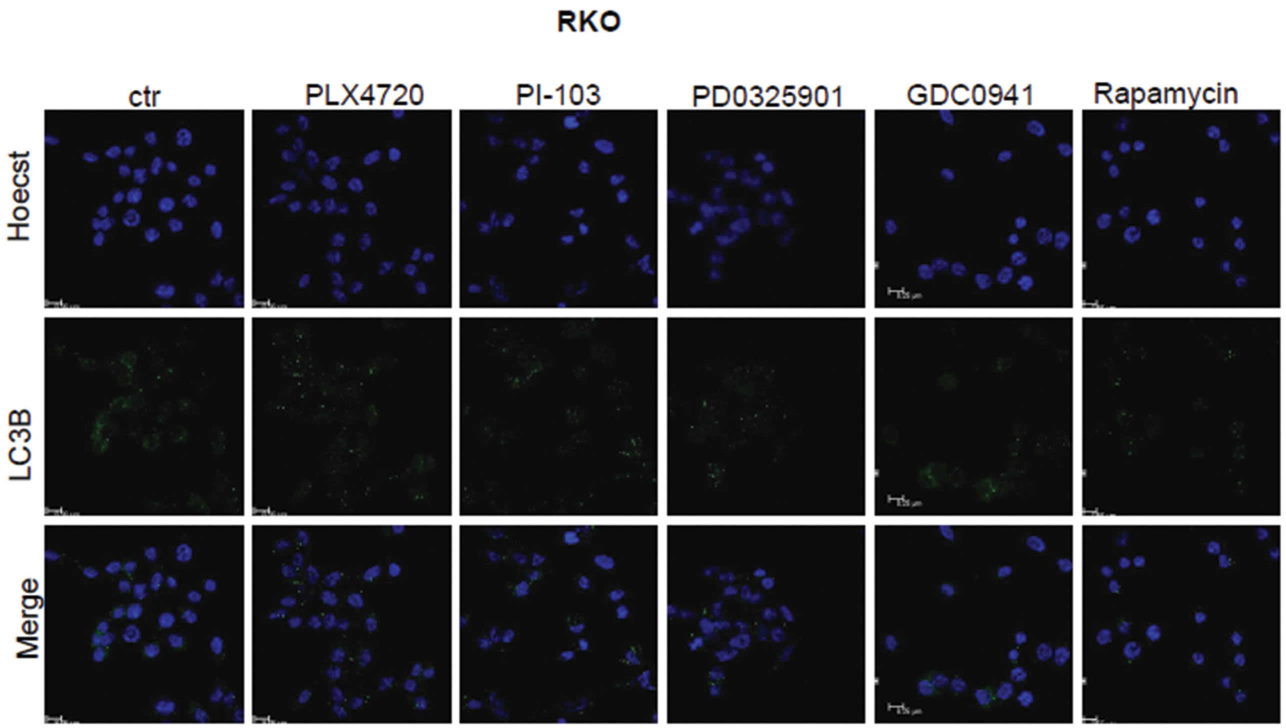

LC3 Staining	ctr	PLX4720	PI-103	PD0325901	GDC0941	Rapamycin
Highly concentrated staining	43 (28.5%)	22 (22%)	20 (33.3%)	14 (14.9)	22 (39.3%)	7 (11.7)
Positive cells	122 (80.8%)	83 (83%)	59 (98.3%)	87 (92.6%)	51 (91.1%)	56 (93.3%)
Broken nuclei		5	12	12		2
Total number of cells	151	100	60	94	56	60

Supplementary Figure S3: Confocal microscope images of two-dimensional culture in RKO cell line after treatment with 1µM of each of the following BRAF/MEK and PI3K/MTOR pathway inhibitors; PLX4720 (2nd column), PI-103 (3rd column), PD0325901 (4th column), GDC0941(5th column) and Rapamycin (6th column) as compared to control untreated cells (1st column). Cells were stained with Hoechst (upper row, blue), LC3 antibody (middle row, green) and merged (lower row). The total number of cells and the number of stained cells from three different confocal images for each sample was recorded.

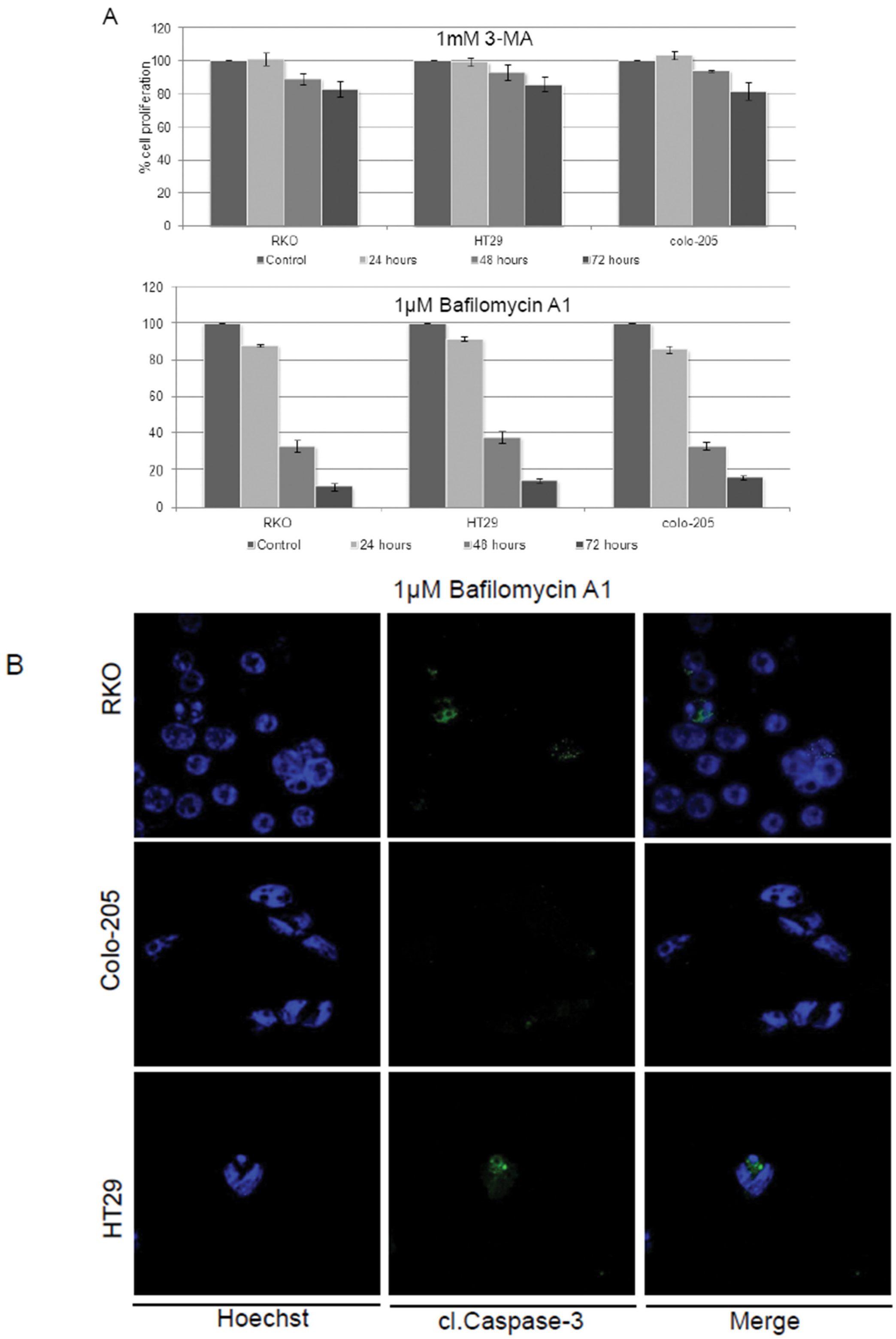

Supplementary Figure S4: A. Cell Viability, using the SRB assay , after treatment with 1mM 3MA and 1µM Bafilomycin A1 (upper and lower graph respectively) for 24, 48 and 72 h in mutBRAFV600E cell lines RKO, Colo-205 and HT29. **B.** Confocal microscope images of two-dimensional culture in RKO, Colo-205 and HT29 after treatment with 1µM Bafilomycin A1 for 48 h. The nuclei were detected with HOECHST staining. Detection of Caspase-3 cleavage was achieved with the use of specific antibody.

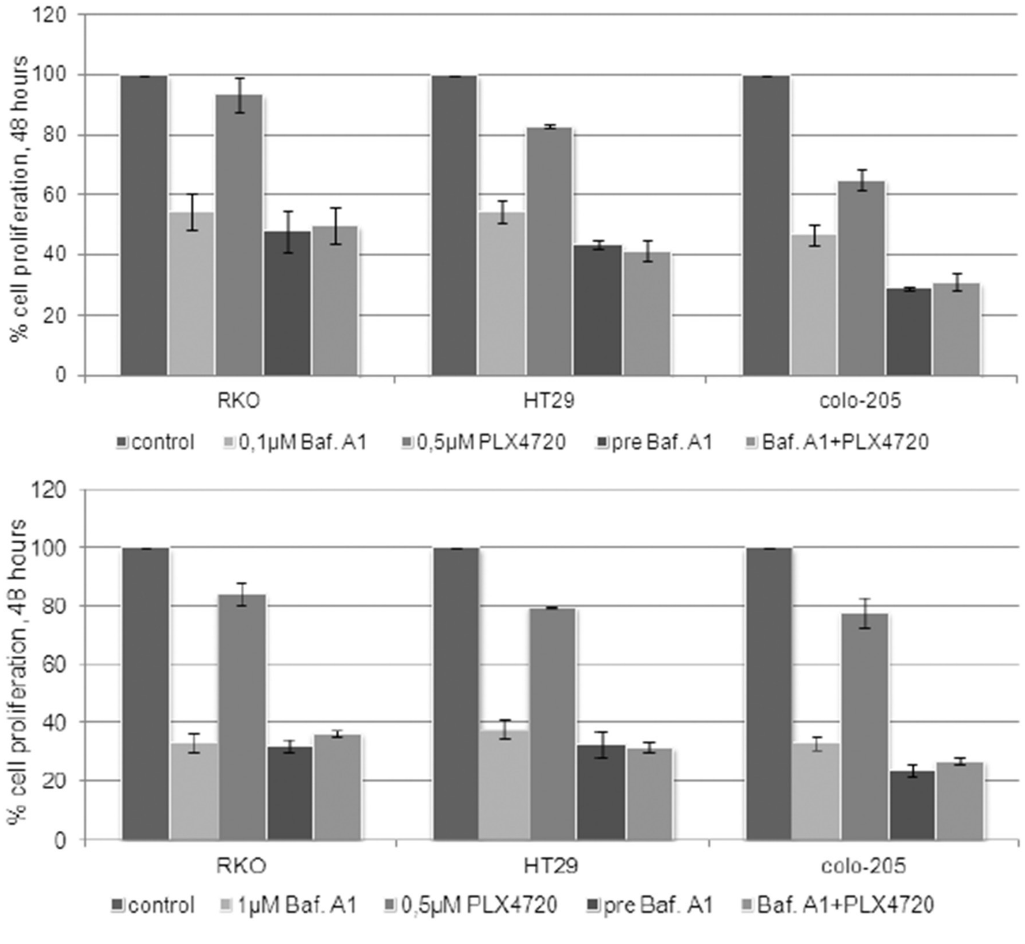

Supplementary Figure S5: Cell viability of BRAFV600E bearing cell lines RKO, Colo-205 and HT29 after mono-, pre- and co-treatment with autophagic and BRAF inhibitors. The cells were treated with 0,5µM PLX4720 and either 0,1µM (upper graph) or 1µM (lower graph) Bafilomycin A1. For the pre-treatment testing, cells were incubated for 2 h with 0,1µM (upper graph) and 1µM (lower graph) autophagic inhibitor Bafilomycin A1 and then co- treated with Bafilomycin A1 and PLX4720.
